# Emergency/Elective Surgery and Emergency Percutaneous Interventions in Liver Hydatid Cysts and Their Results

**DOI:** 10.5152/tjg.2023.22818

**Published:** 2023-10-01

**Authors:** Serdar Öter, Metin Yalçın

**Affiliations:** 1 Department of Gastroenterological Surgery, Manisa City Hospital, Manisa, Turkey; 2 Department of General Surgery, Antalya Training and Research Hospital, Antalya, Turkey

**Keywords:** Hydatid cyst, liver, emergency surgery, emergency percutaneous interventions

## Abstract

**Background/Aims:**

Hydatid cyst may remain asymptomatic for several years or may become complicated. The aim of this study is to evaluate the patients who were operated on for liver hydatid cyst in our clinic and the results of preoperative or postoperative complications.

**Materials and Methods:**

The data of 836 patients who underwent surgery (n = 750) or Puncture, Aspiration, Injection, and Re-aspiration (n = 86) for hydatid cyst disease in our clinic between January 2006 and January 2021 were evaluated retrospectively.

**Results:**

Surgical operation was performed in 750 of the patients and Puncture, Aspiration, Injection, and Re-aspiration procedure was performed in 89 of the patients. In the surgery and Puncture, Aspiration, Injection, and Re-aspiration group, respiratory distress, anaphylaxis, allergic rash, and urticaria were observed in 11 patients (8 in Puncture, Aspiration, Injection, and Re-aspiration group and 3 in open surgery group). All patients recovered with emergency medical interventions. Recurrence was observed after the percutaneous procedure in 11 cases and after surgery in 36 cases. There was no statistically significant difference between the surgical and Puncture, Aspiration, Injection, and Re-aspiration groups in terms of recurrence and cyst infection (*P* = .253 and *P* = .547, respectively). The incidence of the development of intrabiliary rupture, allergic reaction, and intraperitoneal rupture was found 135 (16.14%), 12 (1.43%), and 2 (0.23%) in our study, respectively.

**Conclusions:**

Intraperitoneal or intrabiliary rupture is a rare but fatal complication of hydatid cyst. The presence of fever, jaundice, abdominal pain, urticaria, and anaphylactic reactions in endemic areas should take the suspicion of hydatid cyst rupture. The timing of surgery is an important factor affecting morbidity and mortality. Detailed exploration of the abdomen in emergency surgery for rupture hydatid cyst is essential for recurrence.

**Main Points**

- Intraperitoneal or intrabiliary rupture is a rare but fatal complication of hydatid cyst (HC).
- The timing of surgery of is an important factor for intraperitoneal or intrabiliary rupture of HC.
- The data of 836 patients who underwent surgery (n = 750) or Puncture, Aspiration, Injection, and Re-aspiration (PAIR) (n = 86) for HC were investigated in the text.
- This article is about the results of surgical or interventional treatment of HC and also the results of emergency management for HC.

## Introduction

Hydatid cyst (HC) disease, which is a zoonotic disease, is caused by parasites dependent on the Echinococcus species. Mostly, it is caused by *Echinococcus granulosus* in humans than *Echinococcus multilocularis* which is the causative agent of alveolar echinococcosis.^1^

Asymptomatic patients are usually diagnosed with radiological examinations for other indications.^2^ However, a smaller proportion of patients may become symptomatic after complications such as secondary infections, anaphylaxis, adjacent organ compression, and rupture.^2^ Secondary cyst infections resulting from cystobiliary communication are the most common of them. Increased intra-cystic pressure is a common risk factor for the erosion of adjacent structures due to the growing cyst and the development of complications. 

Hydatid cyst disease is endemic in our country and humans are intermediate hosts. In addition to human and animal health, it maintains its importance as a public health problem in many parts of the world and in our country due to the economic losses it causes.^3-5^

Despite advances in diagnosis and treatment in recent years, eradication has still not been achieved. The retrieved eggs pass through the mucosa of the upper intestinal tract and mix with the portal venous system. It changes to the larval stage in the last organ to which the parasite eggs are attached.^3^

Approximately, 75%-85% of the cysts are localized in the liver.^6^ The second most common organ is the lung with a rate of 20%-30%, while it is seen less frequently in the spleen, kidney, heart, bone, central nervous system, and other organs.^7^

Since cystic echinococcosis grows very slowly, they are asymptomatic for years. The preliminary diagnosis is usually made as a result of radiological examinations performed for other reasons. There are relatively high rates of mortality, morbidity, and recurrence in surgical treatment. The results of medical treatment are not very good.^7,8^ Percutaneous treatment is widely used and minimal morbidity is reported.^9^ Surgery is still the main treatment for HC disease. The aim of the surgical treatment is the elimination of scolexes, the removal of all viable parts of the cyst and the obliteration of the remaining cavity, prevent complications, and recurrence.^10,11^

In the treatment of liver HC, many surgical techniques have been used, from aspiration, drainage, and marsupialization to partial cystotomy—drainage or complete excision of the cyst with segmentary liver resection.^12^ These surgical approaches can be performed laparotomy or laparoscopically.^11^ Rupture of the cyst into the biliary tree is a serious complication occurring in 5%-15% of patients with hepatic involvement and produces a clinical picture of biliary obstruction.^13^

Intraperitoneal rupture may be secondary to spontaneous or traumatic injury and the death ratio due to anaphylaxis in this condition was reported 25%.^14^

Infection of the cyst contents with pyogenic microorganisms is another complication that may be encountered. The process results in the development of abscess formation. It may develop due to fistulas that occur after surgical interventions, percutaneous treatments, or spontaneous rupture of the cyst. This process, which occurs with classical abscess findings (such as abdominal pain, fever, and leukocytosis), is treated with drainage of the abscess and appropriate antibiotics.^13^

The aim of this study is to evaluate the patients who were operated on for liver HC in our clinic and the patients who underwent emergency surgery or emergency percutaneous intervention as a result of preoperative or postoperative complications.

## Materials and Methods

After getting the local ethical committee’s approval, the medical record of patients who were treated for HC in our clinic was retrospectively evaluated between January 2006 and January 2021. All procedures performed in studies involving human participants were in accordance with the ethical standards of the institutional research committee and with the 1964 Declaration of Helsinki and its later amendments or comparable ethical standards. The study was approved by the Bioethics Committee of the Medical Faculty of Harran University (Date: 04/10/2021; Session Number and Decision No: HRU/21.17.01). Informed consent was not obtained from patients because it was a retrospective file data evaluation study.

Demographic data of the patients, presence of accompanying extrahepatic HC, clinical and radiological findings, surgical procedures, length of hospital stay, complications, adjuvant therapy, prolonged bile drainage, cyst infection, per-operative bile duct patency, per-operative anaphylaxis, and recurrence in long-term follow-up were examined.

### Inclusion Criteria

- All patients whose age is more than 18 years with liver HC and treated in our clinic.

### Exclusion Criteria

- Other liver cysts
- Conservatively treated patients
- Patients under 18 years of age
- Patients with missing data

Different methods were used in 750 patients who underwent surgery in our study. These were partial cystectomy (laparoscopic or open), capitonnage, marsupialization, segmentectomy, hepatectomy, pericystectomy, total excision, interflexion, and splenectomy. In the preoperative evaluation, abdominal ultrasonography (USG) examinations were routinely performed and the cysts were staged according to the Gharbi staging system.^14^ Albendazole treatment (10 mg/kg) was started in all patients at least 2 weeks before surgery or percutaneous treatment. Medical treatment was continued for at least 3 months after surgery or percutaneous procedure except for radical surgery. The general trend is that stage I-II cysts should be evaluated primarily for medical treatment and/or percutaneous interventions, and stage 3-4 cysts should be treated surgically.

Since stage 5, cysts were considered inactive, no treatment was applied. 

Puncture, Aspiration, Injection, and Re-aspiration (PAIR) indications; type I, type II, and some type III cysts, some type IV cysts with fluid content, infected cyst or abscess, postoperative recurrence or collection, patients who refuse surgery. In our study, PAİR procedures were performed under operating room conditions, under aseptic conditions, by the same interventional radiologist as sedo-analgesia, laryngeal mask anesthesia, or general anesthesia. As recommended in the 2010 World Healt Organisation - Informal Working Group on Echinococcosis (WHO-IWGE) guidelines, 20% NaCl was used as a scolocidal agent in percutaneous treatment^15^

The disappearance or decrease of the cyst fluid on USG, pseudotumor appearance, semisolid appearance with heterogeneous content, and irregular and thick cyst wall are the known morphological changes in the follow-up showing that the percutaneous treatment is successful. In the follow-up of our percutaneous treatment group, similar findings were considered as cysts that lost viability, and they were called for controls at regular intervals.

In surgical procedures, 20% NaCl or betadine was used. The patients were called for postoperative controls, and hemogram and biochemistry tests were performed. Radiological imaging was also evaluated by the same interventional radiologist.

In the first years of the study, more open operations were performed and in the last years, laparoscopic method and percutaneous treatment were preferred more in suitable patients.

### Statistical Analysis

IBM Statistical Package for Social Sciences version 20.0 (IBM Corporation, Armonk, NY, USA) was used. According to the distribution of normality, Student’s *t*-test and Mann–Whitney *U*-test were used to evaluate the numerical data. Chi-square test was used for the categorical data. Numerical data were given as mean ± standard deviation and median (minimum–maximum values) according to the normality test; categorical values were given as count (n) and percentage (%). A *P* > .05 value was statistically significant. 

## Results

Between 2006 and 2021, 836 patients with the diagnosis of HC were treated in our clinic. Of the patients, 626 were female, 213 were male, and the median age was 48 years (range 18-90). The median cyst size was 67 mm (range: 10-220 mm). While the median number of cysts was 1 (range: 1-10), 253 patients had more than 1 cyst. The most common location of the cysts was the seventh segment (384 patients) of the liver. While only the liver localized cyst was present in 759 patients, 80 patients had HC involvement in other organs (spleen, omentum, kidney, adrenal) as well as the liver, mostly in the lung. Surgery was performed with the diagnosis of pulmonary HC in 37 patients and splenic HC in 27 patients. The most common complaints at admission were abdominal pain (528 patients) and bloating (396 patients). The diagnosis of HC was made by USG and computed tomography.

Surgical operation was performed in 750 of the patients and PAIR was performed in 86 patients. Intraoperative abscess drainage was performed in 51 patients. Postop abscess drainage was performed in 23 cases. The median length of hospital stay was determined as 8 days (range: 1-70). Recurrence was observed in 47 patients during follow-up. 

In the open surgery group, the most performed surgical procedure was open partial cystectomy with 764 cases, including repetitive operations. While marsupialization was added in 25 cases, omentopexy was added in 114 cases and capitonnage was added in 132 cases. Intraoperative biliary fistula was observed in 132 patients. Intraoperative sutures were placed in 125 biliary fistulas in the management of intraoperative biliary fistula. Fistula location could not be determined in 7 patients. Postoperative bile fistula was developed in 32 patients. While 9 of 32 patients who developed postoperative bile fistula recovered with conservative follow-up, postoperative endoscopic retrograde cholangiography (ERCP) and sphincterotomy were performed in 21 patients, percutaneous drains were placed in 1 patient, and ERCP and naso-biliary drains were placed in 1 patient.

In the open surgery group, there was more than 1 cyst in 253 cases. There were 7 cysts in 1 case, 6 cysts in 4 cases, 5 cysts in 9 cases, 4 cysts in 18 cases, 3 cysts in 44 cases, and 2 cysts in 177 cases. In 29 cases, there were intra-abdominal cysts located outside of the lung and liver. Multiple cysts were in the left lobe in 27 cases, in the right lobe in 112 cases, and bilateral in 115 cases. In 2 patients, the intraperitoneal rupture was detected perioperatively. In 7 cases, emergency surgery and abscess drainage in the HC lodge and subhepatic area were performed in the postoperative period. Percutaneous abscess was drained in 16 cases. In the intraoperative period, iatrogenic liver injury occurred in 9 cases, iatrogenic diaphragm injury in 2 cases, iatrogenic stomach injury in 1 case, and iatrogenic ileum injury in 1 case. Mortality was observed in 3 patients in this group. One of these patients died because of myocardial infarction on the postoperative third day. The other 2 patients underwent emergency surgery due to liver abscess, and one died on the sixth postoperative day and the other on the eighth postoperative day due to sepsis and multiorgan dysfunction.

In the surgery group, surgery was started laparoscopically in 79 patients. Conversion to open surgery was performed in 18 cases. Difficulty in reaching the cyst, anatomical localization of the cyst, bleeding, and long learning curve can be counted as the causes of conversion. Laparoscopic partial cystectomy was performed in 57 cases. Three patients underwent laparoscopic peri-cystectomy and 1 patient underwent laparoscopic segmentectomy. Of the patients whose laparoscopic procedure was completed, 11 were male and 50 were female.

In the laparoscopic surgery group, 14 cases are adjacent to the diaphragm. Nine cases were adjacent to the gallbladder. Intra-operative bile duct association was observed in 12 patients. Simultaneous cholecystectomy was performed in 13 cases due to cholelithiasis.

In the intra-operative period, iatrogenic diaphragmatic injury was observed in 2 cases. The diaphragm was repaired laparoscopically in these 2 patients. In 1 case, iatrogenic intestinal injury was observed while suturing the umbilical trocar. The primary suture was performed on the injury area. Mortality was not observed in the laparoscopic surgery group. 

In the laparoscopic surgery group, intra-operative abscess was observed in 3 patients and abscess drainage was performed laparoscopically. In the postoperative period, lodge abscess was observed in 6 cases. A percutaneous drainage catheter was placed in the abdomen for all these patients. In the postoperative period, urticaria was observed in 1 case.

Of 89 patients who underwent PAIR, 71 were female and 18 were male and the median age was 42 years (range 18-90). The PAIR procedure was started in 3 patients, but surgical treatment was performed because the cyst fluid contained bile. (Open surgery was performed in 2 patients and laparoscopy in 1 patient.) The PAIR procedure was performed when recurrence developed in 5 patients who had previously undergone partial cystectomy. Abscess was present in 5 patients. Percutaneous abscess drainage was applied to 5 patients as a second procedure after the PAIR procedure. The percutaneous procedure was performed twice in 3 patients. A percutaneous procedure was performed 3 times in 1 patient. The liver cysts of the patients who underwent the PAIR procedure were located in the right lobe in 57 cases, in the left lobe in 21 cases, and bilateral in 7 cases. Multiple cysts were observed as type 1 cysts in 47 patients, type 2 cysts in 15 patients, type 3 cysts in 5 patients, and type 1, 2, and 4 cysts in 6 patients. The cyst type was not specified in 16 patients. Pleural effusion was observed in 7 patients. Allergic reaction was observed in 6 patients and anaphylactic shock was observed in 2 patients. While PAIR was performed, general anesthesia was applied to 45 patients and sedo-analgesia was applied to 44 patients. No mortality was observed in the PAIR group.

In the surgery and PAIR group, respiratory distress, anaphylaxis, allergic rash, and urticaria were observed in 11 patients (8 patients in the PAIR group, 3 patients in the open surgery group). All patients recovered with emergency medical interventions. 

In the surgery group, atelectasis and pleural effusion were observed after surgery in 11 cases. Respiratory physiotherapy and bronchodilator treatment were applied to these patients and full recovery was achieved in all patients.

Recurrence was observed in 47 patients during follow-up. Recurrence was observed after the percutaneous procedure in 11 cases and after surgery in 36 cases. Abscess healed completely and did not recur after drainage and antibiotic treatment in both groups that underwent surgical or percutaneous drainage for the abscess.

There was no statistically significant difference between the surgical and PAIR groups in terms of recurrence and cyst infection (*P* = .253 and *P* = .547, respectively).

## Discussion

Hydatid cyst is a parasitic infection most commonly caused by a parasite called *Echinococcus granulosus*, causing endemic disease worldwide.^16^ Although the liver and the lung are the most commonly involved organs, hydatid disease may involve any organ in the abdomen, including the spleen, pancreas, kidney, and peritoneal surface.^16^ In our series, 759 patients (90.46%) had only liver HC, while 80 (9.54%) patients had HCs in the lung, spleen, kidney, adrenal, and omentum, mostly in the lung.

There are basically 4 treatment options: surgery, percutaneous technique, antiparasitic medical treatment, and follow-up (wait and see).^17^ The desired goals in the treatment of hepatic hydatid disease include the complete elimination of the parasite and prevention of recurrent disease with minimum morbidity and mortality. Selection of the most appropriate treatment is performed according to the cyst-related features such as the size and number of the cyst, localization, cysto-biliary relationship, and the structure of the cyst, as well as the availability of an experienced surgeon and an experienced radiologist. 

In liver HC, the effectiveness of medical treatment is low and the cure rate is approximately 30%.^7,15^ However, it is an important aid added to these treatment options in preventing unintentional spreads in medical treatment, surgery, and percutaneous treatment.^18,19^ In our study, all patients were taken albendazole treatment at least 2 weeks before the surgical procedure or PAIR procedure. After the treatment, albendazole treatment was continued for at least 3 months.

In uncomplicated cysts, the principle is to inactivate the parasite, remove the germinative membrane, and obliterate the remaining cavity. Although interventional radiological methods can be applied for selected patients (especially Gharbi stages 1 and 2), surgery is the mainstay of treatment.^20^ In our study, the majority of patients were treated with surgery. Different methods were used in 750 patients who underwent surgery in our study. These were partial cystectomy (laparoscopic or open), capitonnage, marsupialization, segmentectomy, hepatectomy, pericystectomy, total excision, interflexion, and splenectomy.

With the increase in the number of laparoscopic interventions as a result of the application of current technology to liver surgery, laparoscopic treatment of cysts located close to the surface has become possible, especially in the inferior-anterior segments that are easy to access.^21,22^ In our study, surgery was started laparoscopically in 79 patients. Conversion to open surgery was performed in 18 cases. Difficulty in reaching the cyst, anatomical localization of the cyst, bleeding, and long learning curve can be counted as the causes of conversion. In our study, the laparoscopic procedure was successfully applied to 61 patients.

Hydatid cysts may remain asymptomatic for several years or may become complicated. Complications of liver HC are rupture into the abdomen, rupture into the bile duct, formation of the liver abscess, and rupture into the pleural space or into the pulmonary parenchyma.^4^ Previous studies reported that intrabiliary rupture (overall: 2%-50%) is the most common complication, allergic reactions (1%-25%) is the second, and intraperitoneal rupture (1%-16%) is the third most common complication of the HC.^1,3^ In our study, the incidence of the development of intrabiliary rupture, allergic reaction, and intraperitoneal rupture are shown in Table 1.

Symptoms that may develop in case of intrabiliary rupture are cholecystitis, cholangitis, obstructive jaundice, peritonitis, pancreatitis, and septicemia.^23,24^ Early diagnosis of intrabiliary rupture of liver HC and early treatment is important because the mortality rate is 50%.^25^ In our study, peritonitis was seen in 4 patients, and obstructive jaundice and cholangitis were seen in 8 patients. Patients with fever, jaundice, and pain in the right upper abdomen in endemic areas should be investigated for intrabiliary rupture of HCs.^12,25^ In our study, intra-operative biliary fistula was observed in 132 patients. Intraoperative sutures were placed in 125 biliary fistulas in the management of intraoperative biliary fistula. In 2 patients, common bile duct exploration and T-tube drainage were added to the surgery, since cyst and major bile duct involvement were observed during the operation. Fistula location could not be determined in 7 patients.

Common symptoms of intraperitoneal rupture of HC are nausea, vomiting, abdominal pain, dyspnea, urticaria, and hemodynamic instability.^13,25,26^ Depending on the amount of HC fluid, it may cause mild reactions to severe anaphylactic shock.^25-27^

Allergic reactions ranging from mild itching to urticaria, skin rash, bronchospasm, and anaphylactic shock may develop before, during, or after the procedure.^24,26^

Anaphylaxis is one of the complications with the highest mortality rate. The most important cause of anaphylaxis is the mixing of the cyst fluid into the circulation. Respiratory distress, anaphylaxis, allergic rash, urticaria, and shock were observed in 11 patients (8 patients performed in pairs, 3 patients in open operation). All patients recovered with emergency medical interventions

Intraoperative abscess drainage was performed in 51 patients. Postop abscess drainage was performed in 23 cases.

Other complications we encountered were pleural effusion, atelectasis, embolism, diaphragmatic injury, liver and spleen bleeding, and wound infection.

While the recurrence rate after conservative surgeries has been reported between 10% and 30%, this rate is less than 5% after radical surgeries.^27-29^ In our study, recurrence was observed in 47 patients during follow-up. Recurrence was observed after the percutaneous procedure in 11 (12.79%) cases and after surgery in 36 (4.80%) cases.

Our study has some limitations. Since it is a retrospective study and a single-center experience, the number of laparoscopic surgery and PAIR group cases is low.

## Conclusion

In conclusion, as the classic open surgical operations laparoscopic surgical operations or PAIR methods are safe and usable in the treatment of the HC disease. It is necessary to choose the treatment according to the patient. The risk of recurrence after both methods should be kept in mind. 

Intraperitoneal or intrabiliary rupture is a rare but fatal complication of HC. Presence of fever, jaundice, abdominal pain, urticarial, and anaphylactic reactions in endemic areas should take the suspicion of HC rupture. Timing of surgery is an important factor affecting to the morbidity and mortality. Detailed exploration of the abdomen in emergency surgery for rupture HC is essential for recurrence.

## Figures and Tables

**Table 1.** Spontaneous Complications of the Hydatid Cyst of the Liver

Complication	Open Surgery Group: 689 Patients (n, %)	Laparoscopic Surgery Group: 61 Patients (n, %)	PAIR Group: 86 Patients (n, %)	Total: 836 Patients (n, %)
Intrabiliary rupture	132 (19.15%)	0	3 (3.48%)	135 (16.14%)
Intraperitoneal rupture	2 (0.29%)	0	0	2 (0.23%)
Allergic reaction	3 (0.43%)	1 (1.63%)	8 (9.30%)	12 (1.43%)
Anaphylaxis	3 (0.43%)	0	8(9.30%)	12 (1.43%)
Liver abscess	51 (7.40%)	3 (4.91%)	0	54 (6.45%)

PAIR, Puncture, Aspiration, Injection, and Re-aspiration.

## References

[1] Akbulut S Parietal complication of the hydatid disease: comprehensive literature review. Med (Baltim). 2018;97(21):e10671. (10.1097/MD.0000000000010671) PMC6392988 29794743

[2] Belli S Akbulut S Erbay G Koçer NE . Spontaneous giant splenic hydatid cyst rupture causing fatal anaphylactic shock: a case report and brief literature review. Turk J Gastroenterol. 2014;25(1):88 91. (10.5152/tjg.2014.3521) 24918138

[3] Tiryaki T Şenel E Akbıyık F Mambet E Livanelioğlu Z Atayurt HF . Kist hidatik hastalıklı çocuklarda on yıllık deneyimimiz. Turk Çocuk Hast Derg. 2008;2:19 25.

[4] Aghajanzadeh M Asgary MR Foumani AA et al. Surgical management of pleural complications of lung and liver hydatid cysts in 34 patients. Int J Life Sci. 2014;8(4):15 19. (10.3126/ijls.v8i4.10893)

[5] Mandal S Mandal MD . Human cystic echinococcosis: epidemiologic, zoonotic, clinical, diagnostic and therapeutic aspects. ASIan Pac J Trop Med. 2012;5(4):253 260. (10.1016/S1995-7645(12)60035-2) 22449514

[6] Karabulut B Bayram G Azılı MN et al. Karaciğer Kist Hidatiğinin Cerrahi ve Perkütan tedavi Sonuçlarının Karşılaştırılması. Turk Çocuk Hast Derg. 2014;3:141 145.

[7] Kapan S Turhan AN Kalayci MU Alis H Aygun E . Albendazole is not effective for primary treatment of hepatic hydatid cysts. J Gastrointest Surg. 2008;12(5):867 871. (10.1007/s11605-007-0458-7) 18085341

[8] Men S Hekimoğlu B Yücesoy C Arda IS Baran I . Percutaneous treatment of hepatic hydatid cysts: an alternative to surgery. AJR Am J Roentgenol. 1999;172(1):83 89. (10.2214/ajr.172.1.9888745) 9888745

[9] Giorgio A Di Sarno A de Stefano G et al. Percutaneous treatment of hydatid liver cyst. Recent Pat Antiinfect Drug Discov. 2009;4(1):29 36. (10.2174/157489109787236274) 19149694

[10] Sayek I Cakmakcı M . Laparoscopic management of echinococcal cysts of the liver. Zentralbl Chir. 1999;124(12):1143 1146. 10670103

[11] Salinas Sedó G Velásquez Hawkins C Saavedra Tafur L . Laparoscopic treatment of the hydatid liver cysts. Rev Gastroenterol Peru. 2001;21(4):306 311. 11818992

[12] Toumi O Ammar H Gupta R et al. Management of liver hydatid cyst with cystobiliary communication and acute cholangitis: a 27-year experience. Eur J Trauma Emerg Surg. 2019;45(6):1115 1119. (10.1007/s00068-018-0995-7) 30191292

[13] Beyrouti MI Beyrouti R Abbes I et al. Acute rupture of hydatid cysts in the peritoneum: 17 cases. Presse Med. 2004;33(6):378 384. (10.1016/s0755-4982(04)98600-9) 15105779

[14] Gharbi HA Hassine W Brauner MW Dupuch K . Ultrasound ­examination of the hydatic liver. Radiology. 1981;139(2):459 463. (10.1148/radiology.139.2.7220891) 7220891

[15] Brunetti E Kern P Vuitton DA Writing Panel for the WHO-IWGE. Expert consensus for the diagnosis and treatment of cystic and alveolar echinococcosis in humans. Acta Trop. 2010;114(1):1 16. (10.1016/j.actatropica.2009.11.001) 19931502

[16] Sayek I Tirnaksiz MB Dogan R . Cystic hydatid disease: current trends in diagnosis and management. Surg Today. 2004;34(12):987 996. (10.1007/s00595-004-2830-5) 15580379

[17] Yagci G Ustunsoz B Kaymakcioglu N et al. Results of surgical, laparoscopic, and percutaneous treatment for hydatid disease of the liver: 10 years experience with 355 patients. World J Surg. 2005;29(12):1670 1679. (10.1007/s00268-005-0058-1) 16311852

[18] Akhan O Ozmen MN Dinçer A Sayek I Göçmen A . Liver hydatid disease: long-term results of percutaneous treatment. Radiology. 1996;198(1):259 264. (10.1148/radiology.198.1.8539390) 8539390

[19] Khuroo MS Dar MY Yattoo GN et al. Percutaneous drainage versus albendazole therapy in hepatic hydatidosis: a prospective, randomized study. Gastroenterology. 1993;104(5):1452 1459. (10.1016/0016-5085(93)90355-g) 8482455

[20] Yorganci K Sayek I . Surgical treatment of hydatid cysts of the liver in the era of percutaneous treatment. Am J Surg. 2002;184(1):63 69. (10.1016/s0002-9610(02)00877-2) 12135724

[21] Alper A Emre A Acarli K Bilge O Ozden I Ariogul O . Laparoscopic treatment of hepatic hydatid disease. J Laparoendosc Surg. 1996;6(1):29 33. (10.1089/lps.1996.6.29) 8919175

[22] Bozan MB Kist Hidatikte Laparoskopik Tedavi. In: Kurt A. (Ed.). Hidatik Kist Hastalığı, İnsanda Hidatid / Hidatik Kist Hastalığı. Ankara, Türkiye: Akademisyen Yayınevi AŞ; 2020:299 309.

[23] Sıkar HE Kaptanoğlu L Kement M . An unusual appearance of complicated hydatid cyst: necrotizing pancreatitis. Ulus Travma Acil Cerrahi Derg. 2017;23(1):81 83. (10.5505/tjtes.2016.26820) 28261778

[24] Ufuk F Duran M . Intrabiliary rupture of hepatic hydatid cyst leading to biliary obstruction, cholangitis, and septicemia. J Emerg Med. 2018;54(1):e15 e17. (10.1016/j.jemermed.2017.09.009) 29107478

[25] Atli M Kama NA Yuksek YN et al. Intrabiliary rupture of a hepatic hydatid cyst: associated clinical factors and proper management. Arch Surg. 2001;136(11):1249-1255. (10.1001/archsurg.136.11.1249) 11695968

[26] Akbulut S Ozdemir F . Intraperitoneal rupture of the hydatid cyst: four case reports and literature review. World J Hepatol. 2019;11(3):318 329. (10.4254/wjh.v11.i3.318) 30967909 PMC6447420

[27] Koç C Akbulut S Şahin TT Tuncer A Yılmaz S . Intraperitoneal rupture of the hydatid cyst disease: single-center experience and literature review. Ulus Travma Acil Cerrahi Derg. 2020;26(5):789 797. (10.14744/tjtes.2020.32223) 32946087

[28] Goja S Saha SK Yadav SK Tiwari A Soin AS . Surgical approaches to hepatic hydatidosis ranging from partial cystectomy to liver transplantation. Ann Hepatobiliary Pancreat Surg. 2018;22(3):208 215. (10.14701/ahbps.2018.22.3.208) 30215042 PMC6125266

[29] Vennarecci G Manfredelli S Guglielmo N Laurenzi A Goletti D Ettorre GM . Major liver resection for recurrent hydatid cyst of the liver after suboptimal treatment. Update Surg. 2016;68(2):179 184. (10.1007/s13304-016-0368-x) 27126358

